# Genome-wide identification and expression analysis of the *NRT* genes in *Ginkgo biloba* under nitrate treatment reveal the potential roles during calluses browning

**DOI:** 10.1186/s12864-023-09732-4

**Published:** 2023-10-23

**Authors:** Jin Feng, Can Zhu, Jiaqi Cao, Chen Liu, Jiaqi Zhang, Fuliang Cao, Xiaohong Zhou

**Affiliations:** 1 State Key Laboratory of Subtropical Silviculture, Zhejiang A&F University, Lin’an, Hangzhou, 311300 Zhejiang China; 2 Co-Innovation Center for Sustainable Forestry in Southern China, College of Forestry, Nanjing Forestry University, Nanjing, 210037 China

**Keywords:** *Ginkgo biloba*, *NRT* genes, Callus browning, Auxin, In vitro culture

## Abstract

Nitrate is a primary nitrogen source for plant growth, and previous studies have indicated a correlation between nitrogen and browning. *Nitrate transporters* (*NRTs*) are crucial in nitrate allocation. Here, we utilized a genome-wide approach to identify and analyze the expression pattern of 74 potential *GbNRTs* under nitrate treatments during calluses browning in Ginkgo, including 68 *NITRATE TRANSPORTER 1* (*NRT1*)*/PEPTIDE TRANSPORTER* (*PTR*) (*NPF*), 4 *NRT2* and 2 *NRT3*. Conserved domains, motifs, phylogeny, and *cis*-acting elements (*CREs*) were analyzed to demonstrate the evolutionary conservation and functional diversity of *GbNRTs*. Our analysis showed that the NPF family was divided into eight branches, with the GbNPF2 and GbNPF6 subfamilies split into three groups. Each *GbNRT* contained 108–214 *CREs* of 19–36 types, especially with binding sites of auxin and transcription factors v-myb avian myeloblastosis viral oncogene homolog (MYB) and basic helix-loop-helix (bHLH). The E_1_X_1_X_2_E_2_R motif had significant variations in GbNPFs, indicating changes in the potential dynamic proton transporting ability. The expression profiles of *GbNRTs* indicated that they may function in regulating nitrate uptake and modulating the signaling of auxin and polyphenols biosynthesis, thereby affecting browning in Ginkgo callus induction. These findings provide a better understanding of the role of *NRTs* during NO_3_^−^ uptake and utilization in vitro culture, which is crucial to prevent browning and develop an efficient regeneration and suspension production system in Ginkgo.

**Supplementary Information:**

The online version contains supplementary material available at 10.1186/s12864-023-09732-4.

## Background

Nitrogen is an essential nutrient for plant growth and development, as it is a building block for proteins, nucleic acids, and other biomolecules [1]. Most plants take up nitrogen mainly in the form of nitrate (NO_3_^−^), which is the most abundant nitrogen source in soil [2]. Plants have evolved sophisticated mechanisms to adjust to different concentrations of NO_3_^−^ acquisition, which is mainly carried out via three models: low-affinity transport system (LATS), high-affinity transport system (HATS), and dual-affinity transport system (DATS) [3–5]. H^+^-ATPases pump NO_3_^−^ across the plasma membrane under electrochemical potential gradients, which is then carried by NITRATE TRANSPORTERS (NRTs) to store in the vacuole or assimilate into amino acid in the cytoplasm.

Nitrate transporters are divided into four families, NITRATE TRANSPORTER 1 (NRT1)/PEPTIDE TRANSPORTER (PTR) (NPF), NITRATE TRANSPORTER 2 / Nitrate-Nitrite Porter (NNP) (NRT2) [6], CHLORIDE CHANNEL (CLC) [7], and the SLOW ANION ASSOCIATED CHANNEL HOMOLOG (SLAC/SLAH) [8]. The NPF and NRT2 families were involved in the primary nitrate reaction [9]. NPF is also known as SOLUTE CARRIER 15 (SLC15), PEPTIDE TRANSPORTER (PepT/PTR), or PROTON-COUPLED OLIGOPEPTIDE TRANSPORTER (POT) [10]. The *NPF* family in plants is highly homologous in sequence but diverse in functions. Due to its diverse substrate selectivity, NPF can transport protons, peptides, and various nitrogenous organic solutes [11–14]. Léran [10] compared the phylogeny of the NPF family in 33 fully sequenced plant genomes and found that the original NRT1 family clustered into the NPF1-NPF7 subfamilies, while the PTR family grouped in the NPF8 subfamily. NRT2 is a two-component nitrate uptake system requiring the additional NITRATE ASSIMILATION-RELATED PROTEIN 2 (NAR2), also known as NRT3 [15]. NRT2 and NAR2 are closely clustered as nitrate-associated genes [16], forming functional units to maintain NRT2 plasma membrane targeting and protein stability [14]. However, not all NRT2 require NAR2, such as AtNRT2.4 [17], OsNRT2.3b [18], and OsNRT2.4 [19]. Previous studies have shown that NPF is primarily responsible for LATS, while NRT2 is primarily responsible for HATS. They operate at nitrate concentrations above or below 1 mM [20]. For example, AtNPF2.12/NRT1.9, which is involved in early embryonic development, is a low-affinity nitrate transporter [21]. However, NIP/LATD from *Medicago truncatula*, classified as NPF1, was a nitrate transporter with high affinity [22]. AtNPF6.3/NRT1.1 has been proven to belong to dual-affinity nitrate transporters [6]. Moreover, the dual-affinity nitrate transporters could be switched by phosphorylation of the key residue Thr101 [23]. *MtNPF6.8 *[24] and *OsNRT2.4 *[19] have been confirmed to encode proteins in *Xenopus laevis* oocytes as dual-affinity nitrate transporters. Therefore, identifying NPF and NRT2 as LATS and HATS is complicated, and the function of each member needs further research and verification.

Browning is one of the major issues in woody plant regeneration in vitro, as more phenolic compounds may accumulate during lignin biosynthesis. Numerous studies have revealed a link between browning and nitrogen. Reduction of NO_3_^−^ in the medium could inhibit browning during callus induction in *Bacopa monnieri* [25]. In *Paeonia suffruticosa,* callus browning was also well controlled when the basal salt changed from MS to 1/4 × MS [26]. Daigen et al. (2000) revealed that reducing the content of KNO_3_ and (NH_4_)_2_SO_4_ in the medium can significantly reduce the browning degree in rice [27]. Chopin et al. found that the seed coat of *NRT2.7* mutants was brown [28]. Cristóbal investigated the potential link between N and browning in sweet cherries after harvest. The study found that the phenolic composition and the oxidative states of the cherries, which were influenced by N treatment, could affect the fruit’s post-harvest shelf life [29].

Ginkgo is a gymnosperm species whose callus tends to brown within three months [30]. The transgenic hairy roots were also susceptible to browning due to slow growth [31]. It is essential to prevent browning to develop a highly efficient regeneration and suspension production system in Ginkgo. We treated calluses with various concentrations of KNO_3_ and found that browning was more severe in higher concentrations. Nitrogen is the primary regulator of this process, so we investigated the role of NRTs during nitrogen treatment. To better understand the relationship between NRTs and callus browning, we identified the NRTs gene family in the Ginkgo genome and systematically analyzed the physicochemical properties, conserved domains, and conserved motifs of GbNRTs proteins. We also analyzed the gene structure, phylogenetic relationships between genes, replication patterns, election pressures, *cis-acting elements*(*CREs*), and transcription factor binding sites of the promoter fraction. Furthermore, we detected the expression of *GbNRTs* under different nitrate concentration treatments by RNA-seq and Quantitative Real-Time PCR (qRT-PCR) assay during calluses induction. This study may be the first step in understanding the molecular mechanism of NO_3_^−^ uptake and utilization in the process of Ginkgo tissue culture and identifying the gene family associated with NO_3_^−^ transport in *Ginkgo biloba*. In conclusion, understanding the role of NRTs during NO_3_^−^ uptake and utilization in vitro culture and identifying the genes associated with NO_3_^−^ transport is crucial to prevent browning and develop a highly efficient regeneration and suspension production system in Ginkgo.

## Results

### Genome-wide NRT protein characterization in *Ginkgo biloba*

To identify all *NRTs* members in Ginkgo, the Hidden Markov Model (HMM) of PTR2 (PF00854), MSF_1 (PF07690) and NAR2 (PF16974) were used to screen the *G. biloba* database. A total of 74 GbNRT proteins were identified, including 68 NPFs, 4 NRT2s, and 2 NRT3s (Table S1). The number of amino acids (aa) within the NPF and NRT2 subfamilies exhibit a range of 366 (GbNPF6.4) to 797 (GbNPF5.14), while their corresponding molecular weights span from 41.15 kDa to 88.76 kDa (Table S2). In contrast, proteins of the NRT3 subfamily are notably shorter (200 ~ 300 aa) and smaller (23.35 kDa ~ 32.98 kDa). The isoelectric point (pI) of NRTs ranged from 5.97 to 9.51 and the pI value of most members (10/74) was more than 7. In addition, most GbNRTs are hydrophobic, but 2 GbNRT3s are hydrophilic with negative GRAVY values. The number of transmembrane domains (TMs) in NPF and NRT2 proteins is between 9 ~ 12, except NRT3 protein has only 2 TMs. In terms of subcellular localization, most of the NRT family is localized in the plasma membrane; only GbNPF3.1, GbNPF3.4, and GbNPF3.5 in the nucleus, whereas GbNRT3.1 in chloroplasts.

### Phylogenetic analysis of GbNRTs

**Fig. 1:**
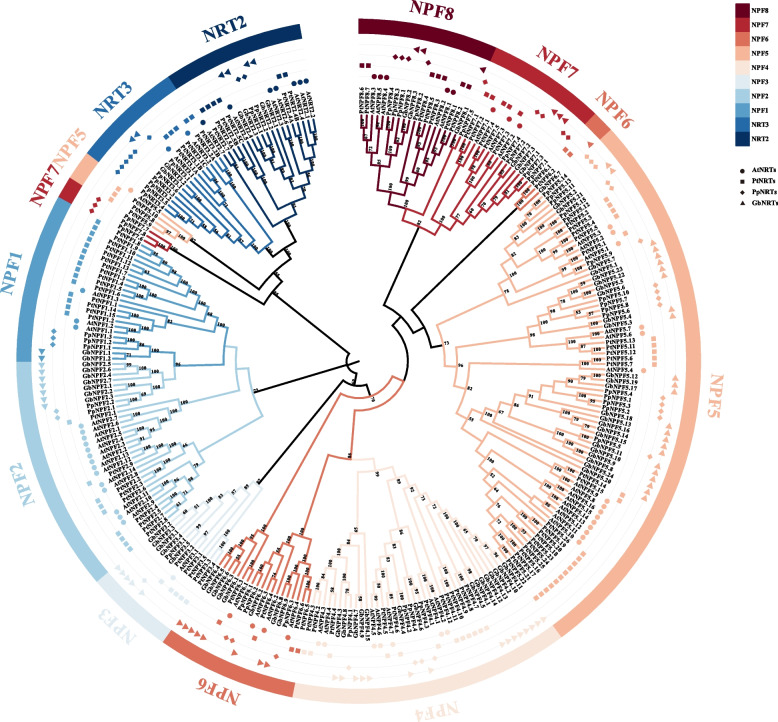
Phylogenetic analysis of NRTs proteins from *A. thaliana*, *P. trichocarpa*, *P. pinaster*, and *G. biloba*. The Neighbor-joining phylogenetic tree was constructed by Molecular Evolutionary Genetics Analysis (MEGA 7.0) with 1000 bootstrap replicates using a total of 246 proteins sequences, including 62 from *A. thaliana* (circles), 96 from* P. trichocarpa* (square), 58 from *P. pinaster* (diamond), and 74 from *G. biloba* (strangle)

A neighbor-joining phylogenetic tree was constructed with 62 NRTs in *A. thaliana*, 96 NRTs in *Populus trichocarpa*, and 58 NRTs in *Pinus pinaster* (Fig. 1). The NRTs of Ginkgo were clustered more closely with Pinus pinaster as expected. The NRT2 and NRT3 subfamilies included fewer members compared to the NPF subfamily. The GbNPF subfamily, with 68 members, was further divided into eight branches and named accordingly from GbNPF1.X to GbNPF8.X based on Léran et al. [10]. GbNPF1 was evolutionarily close to GbNPF2. GbNPF5 had 24 members, followed by GbNPF4 with 15, GbNPF6 with 9, and GbNPF2 with 7 members. GbNPF1, GbNPF7, and GbNPF8 consist of fewer genes in comparison to the other GbNPFs. Notably, In the GbNPF8 branch, nine genes were excluded from the phylogenetic tree due to domain deletions. This observation highlights a clear trend of ongoing degeneration and functional decline within the PTR family in Ginkgo. In addition, the AtNPF2, AtNPF5, and AtNPF6 subfamilies were divided into two groups, while the PtNPF2 and PtNPF5 subfamilies were divided into two groups, and the PtNPF6 subfamilies were divided into three groups. The PpNPF6 and PpNPF7 subfamilies were divided into two groups, and the GbNPF2 and GbNPF6 subfamilies were divided into three groups.

### Motifs, Conserved Domain, and Gene Structure analysis of GbNRTs

**Fig. 2:**
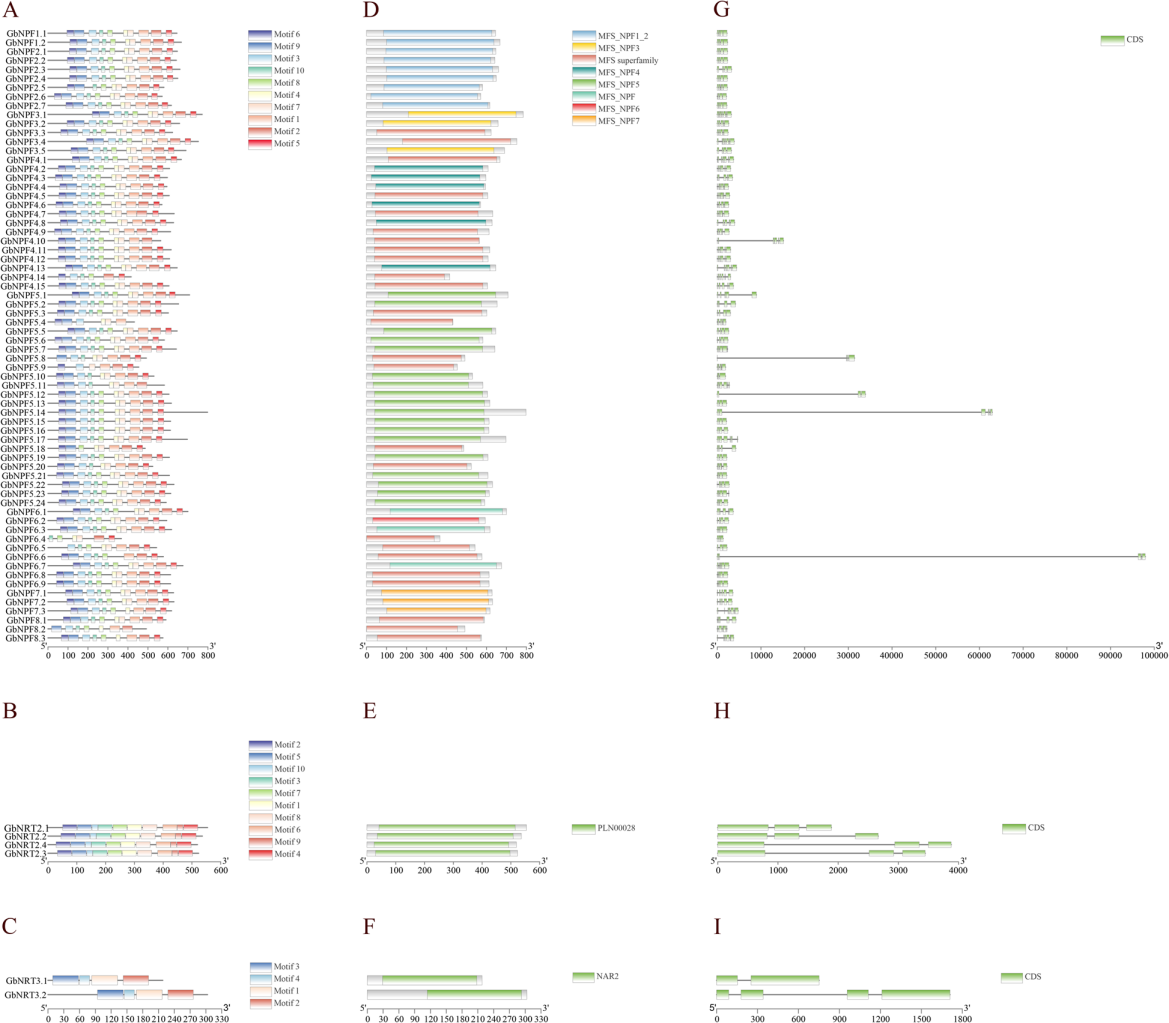
Motif, conserved domain, and gene structure analysis of GbNRTs. **A**-**C** Conserved motifs in GbNRT protein sequences. Ten distinct motifs were identified. **A** Motifs in GbNPF proteins, **B** Motifs in GbNRT2 proteins, and (**C**) Motifs in GbNRT3 proteins. Detailed sequence information for these motifs is provided in supplemental Table S3 (**D**-**F**) Conserved domains within GbNRT protein sequences. **D** Conserved domains in GbNPF proteins, **E** Conserved domains in GbNRT2 proteins, and (**F**) Conserved domains in GbNRT3 proteins. **G**-**I** Gene structure analysis of GbNRT protein sequences. The coding sequences are represented by green boxes, while introns are indicated by gray lines. **G** Gene structure of GbNPF proteins, (H) Gene structure of GbNRT2 proteins, and (**I**) Gene structure of GbNRT3 proteins

Various features, such as conserved motifs, gene structure, and conserved domains can reflect the level of gene family conservation. In Ginkgo, the NPF subfamily is characterized by the presence of the Plant NRT1/PTR family (NPF) domain, which belongs to the Major Facilitator Superfamily of transporters (MFS_NPF) (Fig. 2D). The MFS_NPF is a branch of the proton-coupled oligopeptide transporter (POT/PTR) family of transporters (MFS_POT), which is known to share highly conserved E_1_X_1_X_2_E_2_R/K, PTR2-1, and PTR2-2 motif across various species. However, in Ginkgo, only two motifs, E_1_X_1_X_2_E_2_R/K and PTR2-1 NPF, were detected. The E_1_X_1_X_2_E_2_R motif was complete in 36 GbNPFs but needed to be included or completed in 32 GbNPFs. The PTR2-1 motif in GbNPFs changed slightly compared to other organisms (Fig S1). Using the MEME program, ten conserved motifs were predicted, with most GbNPFs (76.47%) containing all 10 motifs, while a few lacked 1–4 motifs (Fig. 2A). Regarding gene structure, GbNPFs had 3–7 exons (Fig. 2G). In contrast, the GbNRT2 subfamily was highly conserved with 10 conserved motifs (Fig. 2B), with a PLN00028 domain (Fig. 2E), and 3 exome regions (Fig. 2H). The GbNRT3 protein was much shorter, with only 4 highly conserved motifs (Fig. 2C), with an NAR2 (PF16974) domain (Fig. 2F). and the exons were two in GbNRT3.1 and four in GbNRT3.2 (Fig. 2I).

### The regulatory mechanism of the promoter region of *GbNRTs*

**Fig. 3:**
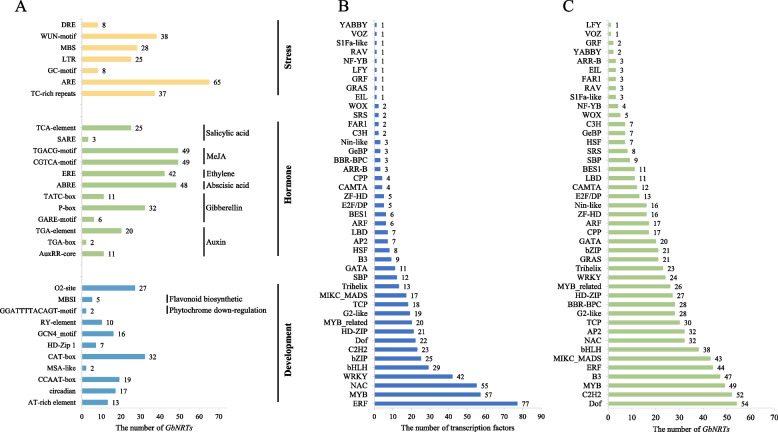
Analysis of cis-acting regulatory elements (CREs) and transcription factor (TF) binding sites in the GbNRT promoters. **A** Distribution of CREs in the promoter regions of GbNRTs. The X-axis represents the count of GbNRTs with these specific CREs in their promoter regions. **B** Number of transcription factors per family capable of binding to the identified binding sites in GbNRT promoter regions. **C** Number of GbNRTs hosting binding sites for specific transcription factor families in their promoter regions

The analysis of *cis*-acting regulatory elements (*CREs*) of *GbNRTs* was conducted to gain further insights into the potential functions of the gene family. A total of 104 *CREs* were identified from the promoters of 74 *GbNRTs* and were broadly categorized into three groups: stress, hormone, and development (Fig. 3A). The stress response elements included MYB binding site involved in drought-inducibility (MBS), anaerobic induction (ARE, GC-motif), low-temperature responsiveness (LTR), Stress and wound-responsive (TC-rich repeats, DRE, WUN-motif). The growth and development regulatory factors comprised cis-acting regulatory element involved in circadian control (circadian), meristem expression (CAT-box), seed-specific regulation (RY-element), differentiation of the palisade mesophyll cells (HD-Zip 1), endosperm expression (GCN4_motif), cell cycle regulation (MSA-like). The hormone response elements included auxin-responsive element (AuxRR-core, TGA-box, TGA-element), gibberellin-responsive element (GARE-motif, P-box), abscisic acid responsiveness (ABRE), ethylene (ERE), salicylic acid responsiveness (SARE, TCA-element), MeJA-responsiveness (CGTCA-motif, TGACG-motif). The promoter region of each *GbNRT* contained 108–214 *CREs* of 19–36 types (Table S4).

Based on the prediction analysis of potential transcription factors (TFs) that can bind to *GbNPF* promoters, 551 TF genes belonging to 43 TF families were found to have potential binding sites in the promoter region of *GbNPFs*. The *ERF* family was the most abundant with 77 members, followed by *MYB* (56), *NAC* (55), *WRKY* (42), as well as other TF gene families (Fig. 3B). Interestingly, some TF families had binding sites in the promoter regions of most of the 74 *GbNRTs*. The *Dof* family had the highest frequency with 54 of the 74 *GbNRTs* promoter regions having potential target binding sites, followed by *C2H2* (52), *MYB* (49), *B3* (47), *ERF* (44), *MIKC_MADS* (43), *bHLH* (38), *NAC* (32), and *AP2* (32) (Fig. 3C).

### Chromosome Distribution of *GbNRTs*

**Fig. 4:**
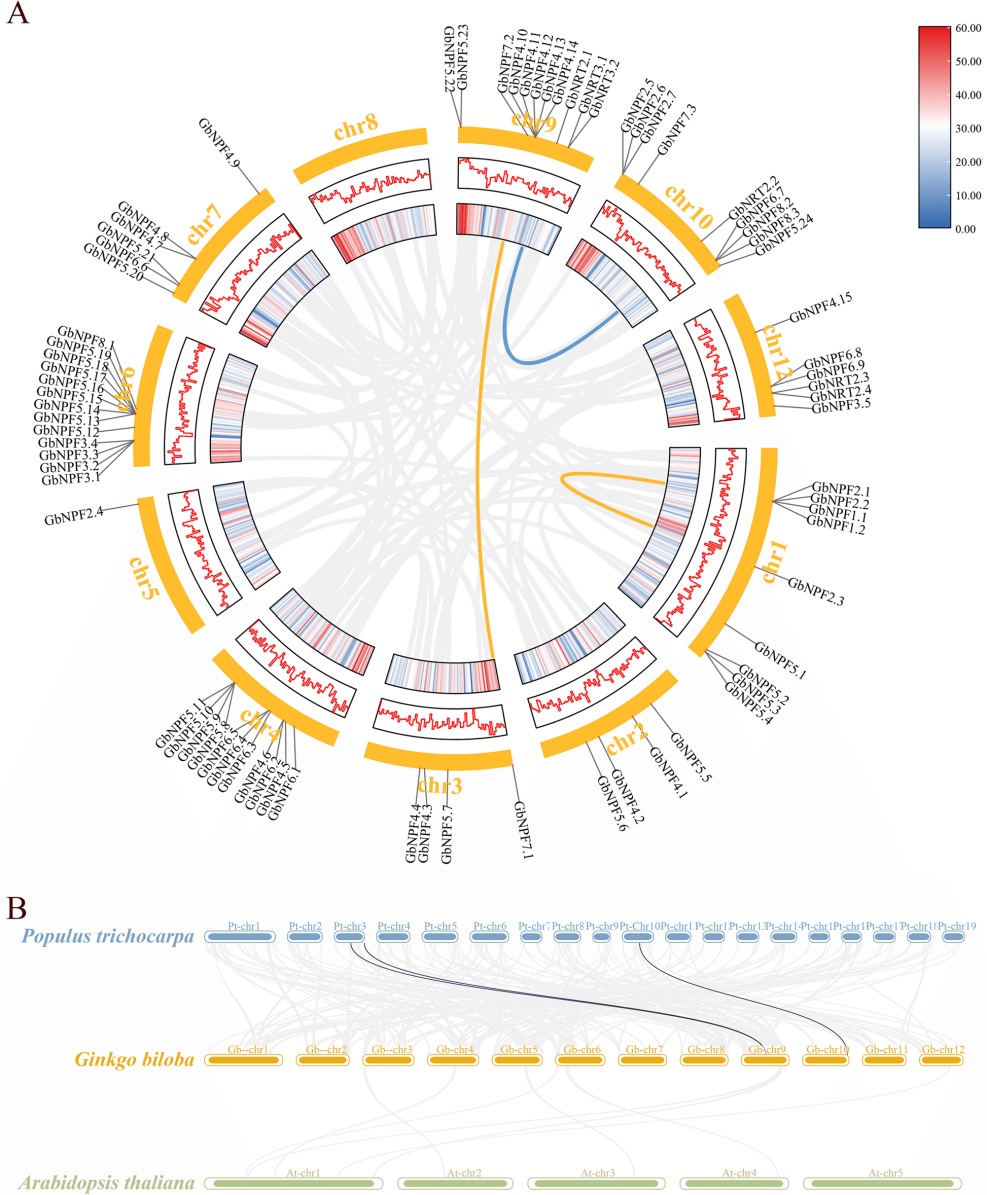
Expansion and evolution analysis of GbNRTs in G. biloba. **A** Chromosomal location and collinearity analysis of GbNRTs genes in the genome of G. biloba. The background circle features gray lines representing colinear gene pairs within the Ginkgo genome. The yellow line signifies a colinear gene pair within the NPF family, while the blue line represents a colinear gene pair within the NRT2 family. The inner circle, denoted by blue and red boxes, illustrates the gene distribution density across chromosomes. The middle circle similarly indicates gene density through a polyline. Chromosome numbers are displayed outside the yellow circle. **B** Synteny analysis of NRTs between G. biloba and A. thaliana and P. trichocarpa. The background showcases gray lines denoting collinearity among different G. biloba and the two other species. Meanwhile, black lines symbolize paralogous NRT gene pairs

The chromosome location of each gene was analyzed based on Ginkgo genome annotation [32]. The 74 *GbNRTs* were found to be unevenly distributed on 10 of the 12 chromosomes, most located near the two ends of chromosomes and fewer genes in the middle (Fig. 4A and Fig S2). Notably, there were no *GbNRTs* on Chr 8 and Chr 11, and only one *GbNRT* on Chr5. All *GbNPF1* members were found to be located on Chr1, while the *GbNPF3* group preferred to distribute on Chr6. Five of the 9 *GbNPF6* members were located on Chr4, and the 3 *GbNPF7* members were located on different chromosomes. The *GbNPF4* and *GbNPF5* groups were large and distributed on 5–6 chromosomes. Additionally, *GbNRT 2* and *GbNRT3* were located on shorter chromosomes, with *GbNRT 2* being evenly distributed on Chr9, Chr10, Chr12, and *GbNRT3* being located on Chr9.

### Amplification and evolution of *GbNRTs*

The gene duplication events and collinearity were analyzed within and between species to further investigate the amplification and evolution process within the *NRT* family. Among the *GbNRTs*, 82% (18/22) of the gene pairs had a Ka/Ks ratio below 0.5, indicating that these *GbNRTs* genes underwent purifying selection during evolution. Additionally, nine tandem repeats containing 19 *GbNRTs* and three paralogs were identified (Fig. 4A and Table S5). Notably, due to the formula’s limitation, the Ks value of the *GbNRT2.1*/*GbNRT2.2* gene pair could not be calculated. It is speculated that these homologous gene pair have undergone significant mutations, resulting in different functions. From a biological perspective, it is understood that most sites with the potential for synonymous mutations have already undergone such mutations, leading to a significant sequence divergence and considerable evolutionary distance between the two genes.

Collinearity analysis revealed that no homologous genes were detected between *GbNRTs* and *AtNRTs*, but three pairs of orthologous genes were identified between *GbNRTs* and *PtNRTs*. The Ka/Ks values of most orthologous genes were smaller than those of paralogous genes, indicating that *NRT* orthologous underwent stronger selection pressure than paralogs during molecular evolution, resulting in a slower and more conservative evolution process (Fig. 4B).

The Ginkgo encoding gene underwent relatively fewer evolutionary replication events, with only 774 pairs of segmental duplication gene pairs and 3,116 pairs of tandem duplicated gene pairs. In comparison, *A. thaliana* had 4,426 pairs of segmental duplication gene pairs and 2,098 pairs of tandem duplicated gene pairs, while *P. trichocarpa* had 15,487 segmental duplication gene pairs and 2,626 tandem duplicated gene pairs (Fig S3). These findings indicated that tandem replication events are the main mode of gene replication in the *GbNRT* family instead of segmental duplication.

### Expression profiles of *GbNRTs* under nitrate treatment

**Fig. 5:**
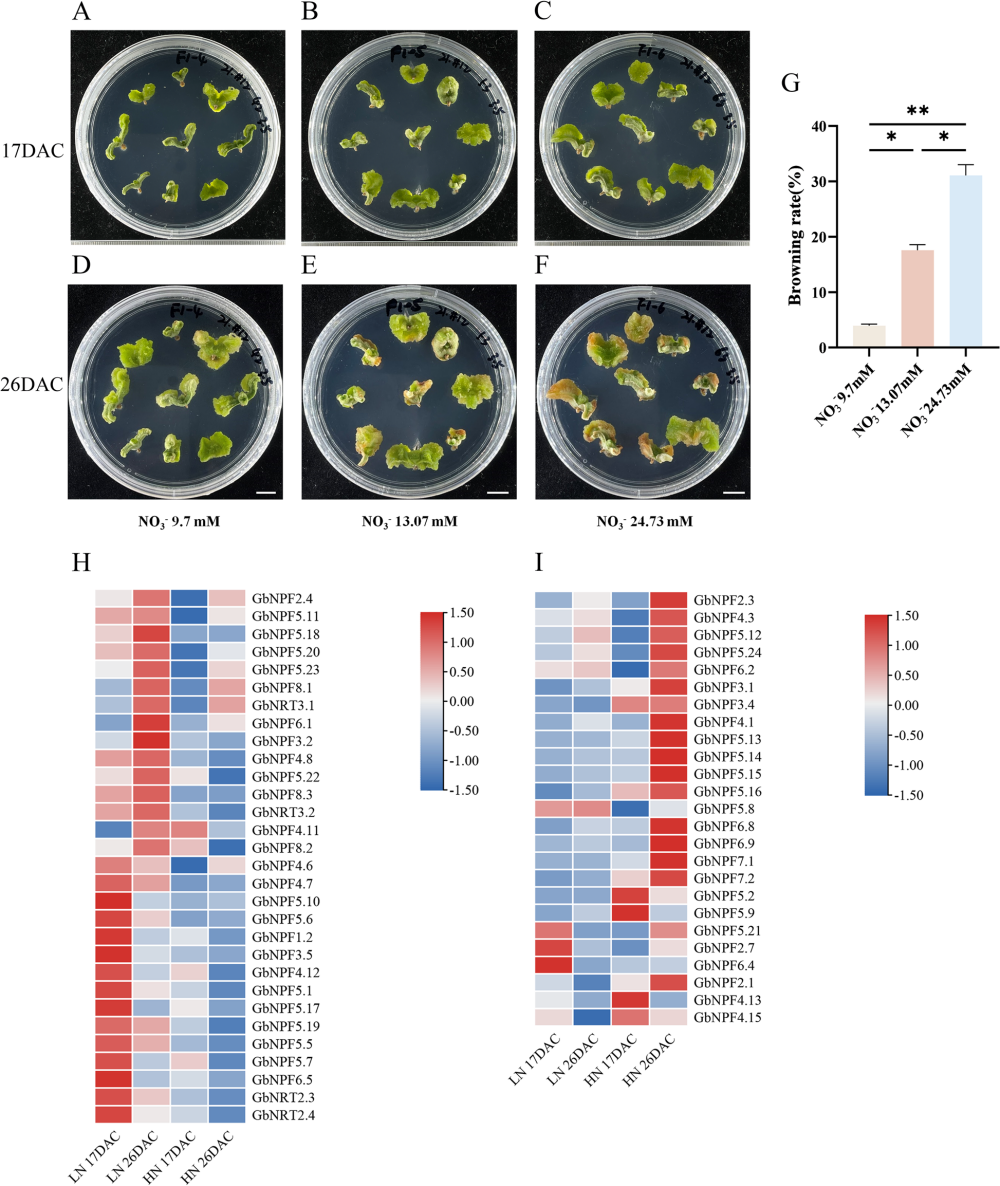
Callus browning and transcriptional expression of GbNRTs in Ginkgo under different nitrate treatments. **A**-**C** Calluses induced from zygotic embryos under different nitrate treatments for 17 days. **D**-**F** Calluses induced from zygotic embryos under different nitrate treatments for 26 days. **G** Browning rate of Ginkgo callus under nitrate treatments. Callus browning was evaluated 26 days after treatments. Data are represented as mean ± SD (*n* = 30, three biological replicates). Results were analyzed using one-way ANOVA for each treatment (* *p* < 0.05, ** *p* < 0.01). **H**-**I** Heatmap of relative expression of 55 GbNRTs under nitrate treatments via RNA-seq. **H** IB GbNRTs, **I** PB GbNRTs

**Fig. 6:**
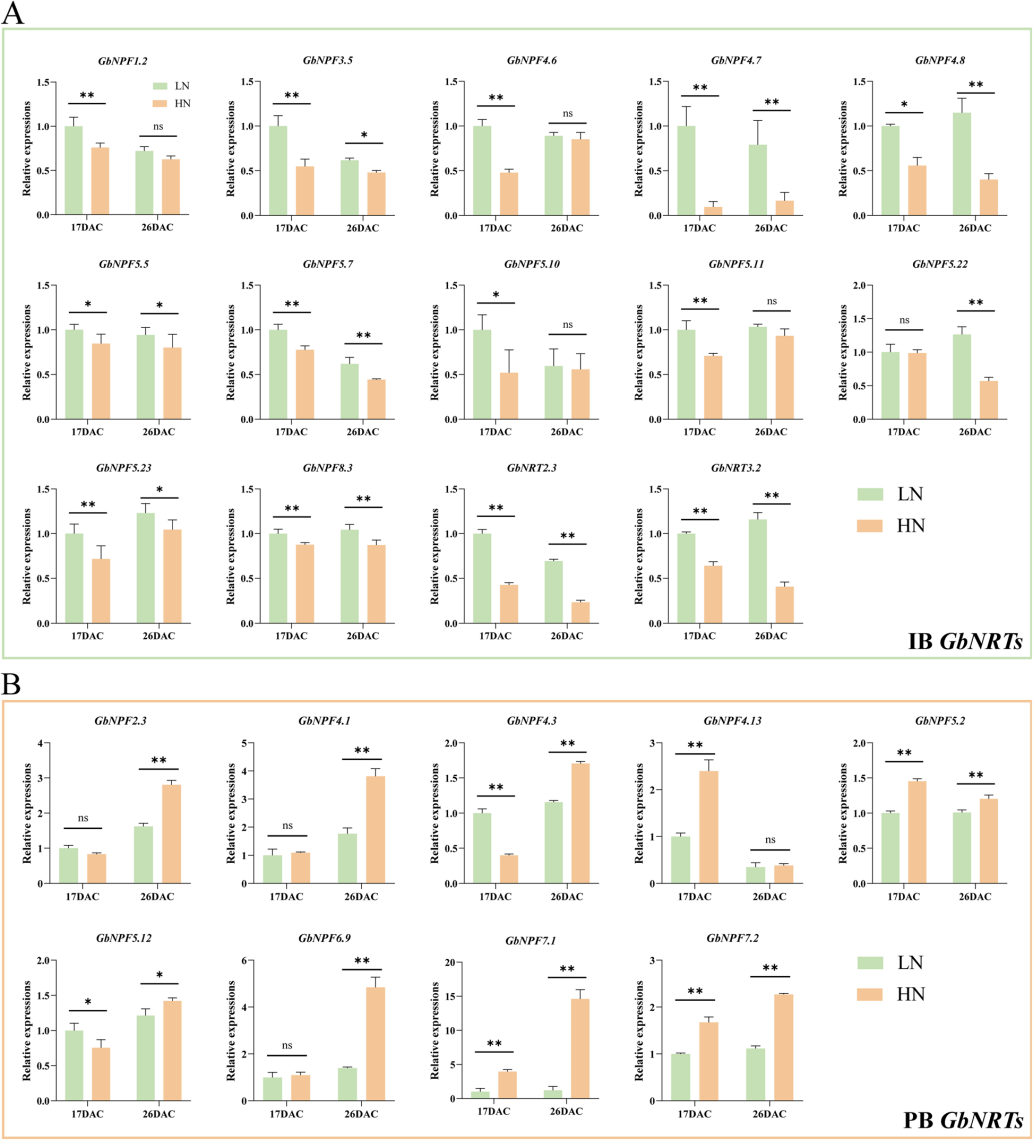
Expression of GbNRTs during callus browning under nitrate treatments at 17 DAC and 26 DAC. Twenty-three GbNRTs (14 IB GbNRTs (**A**) and 9 PB GbNRTs (**B**) were selected for RT-qPCR, each performed with three biological replicates. Data are presented as means ± SD and were normalized by the reference gene GbUBQ. LN treatments are represented by green boxes, while HN treatments are depicted by yellow boxes. Statistical analysis was performed using one-way ANOVA for each treatment (* *p* < 0.05, ** *p* < 0.01)

Previous studies have exhibited the potential link between nitrate and browning in vitro culture [33, 34]. In this study, we induced calluses of Ginkgo with different nitrate concentrations (9.7 mM as low nitrate (LN) condition, 13.07 mM and 24.73 mM as high nitrate (HN) condition). Browning of calluses was almost invisible after 17 days, yet the extent of browning obviously fluctuated up until 26 days (Fig. 5A-G). Notably, most of the calluses treated with 24.73 mM NO_3_^−^ displayed a distinct brown coloration (Fig. 5F). We collected calluses treated with NO_3_^−^ (9.7 mM and 24.73 mM) for 17 days and 26 days to detect the expression of *GbNRTs* by RNA-seq and qRT-PCR. The expression profile revealed that of the 74 *GbNRTs*, 25.68% were not expressed, while the remaining 55 *GbNRTs* showed differential expression patterns that could be broadly divided into 2 categories (Fig. 5H-I and Table S6). Notably, the expression levels of 30 *GbNRTs* were significantly higher with LN treatment than with HN treatment. These *GbNRTs* were defined as inhibiting browning *GbNRTs* (IB *GbNRTs*) (Fig. 5H). The remaining 25 *GbNRTs* were highly expressed with HN treatment, such as the NPF7 subfamily. These *GbNRTs* were classified as promoting browning *GbNRTs* (PB *GbNRTs*) (Fig. 5I). Twenty-three *GbNRTs* were differentially expressed at 17 days or 26 days after NO_3_^−^ treatment and significantly correlated with browning (Fig. 6). Most IB *GbNRTs* exhibited differential expression at both the onset (17 DAC) and later stage (26 DAC) of browning under LN and HN conditions (Fig. 6A). Among these *GbNRTs*, *GbNPF1.2*, *GbNPF5.10*, and *GbNPF5.11* only displayed different expressions at 17 DAC, and *GbNPF5.12* displayed a different expression at 26 DAC (Fig. 6A). For PB *GbNRTs*, only *GbNPF4.13* exhibited differential expression at 17 DAC, while *GbNPF2.3*, *GbNPF4.1*, and *GbNPF6.9* exhibited differential expression at 26 DAC*.* The other PB *GbNRTs* were significantly higher at 17 DAC and 26 DAC under HN condition than LN condition (Fig. 6B). The expression patterns of *GbNRTs* varied during browning under LN and HN conditions, suggesting the potential diverse roles of *GbNRTs* in the browning process.

## Discussion

### Unveiling *NRTs* in *G. biloba*: phylogenetic insights and evolutionary significance

Nitrate serves as the primary nitrogen source for most plants, and *NRTs* are responsible for nitrate allocation across a wide range of NO_3_^−^ concentrations [35]. While previous studies primarily focused on angiosperms, our investigation shines a light on the unique NRT landscape in Ginkgo, an evolutionarily distinct gymnosperm [36]. This study identified 74 GbNRT proteins, including 68 NPFs, 4 NRT2s, and 2 NRT3s. NPF and NRT2 have been shown to co-code the NRT gene family, but they are two distinct families with high sequence similarity [37]. NRT3/NAR2 is a chaperone protein to assist NRT2 in nitrate transport.

The classification of the NPF subfamily has been a matter of ongoing discussion, with differing approaches proposed by various researchers. Wittgenstein [38] divided the NPF family into 10 supergroups based on the phylogeny of NPF from 20 plant species. In this study, we follow the classification proposed by Léran et al. [10], which split the NPF family into 8 subfamilies across various species. However, our analysis found interesting patterns that challenge this classification. Notably, subfamilies previously considered homogenous, such as AtNPF2, AtNPF5, and AtNPF6, showed subdivision in our analysis. Similar patterns emerged in PtNPF2 and PtNPF5, as well as PpNPF6 and PpNPF7, indicating a need for revised classification methodologies. Other studies have seen a similar trend. For instance, when characterizing the genome-wide profile of the NPF gene family in *Malus* × *domestica* Borkh., 15 MdNPF2 were further divided into two groups [39]. Similarly, In *Brassica napus*, BnNPF2, BnNPF5, and BnNPF6 were all divided into two groups [40], as well as CsNPF2, CsNPF5, and CsNPF6 in *Camellia sinensis* [41].

### Key structural features of the GbNPF family indicated dynamic proton transporting ability

Analyzing the conserved motifs of the NPF family uncovers crucial structural features influencing proton transport across cell membranes. NPF has three relatively conserved motifs that vary among different members and across different plants [39]. The E_1_X_1_X_2_E_2_R motif, with its distinct variations in the NPF family, plays a critical role in proton coupling and active transport. The "X" residues within this motif exert an influence on the electrochemical gradient, thereby affecting the transport function of NPF proteins [42, 43].

In the GbNPF4 subfamily, most members have a complete E_1_X_1_X_2_E_2_[R/K] motif, but 32 members lack or have an incomplete motif. Notably, GbNPF4 and GbNPF7 deviate from this pattern. In the GbNPF4 subfamily, most members retain only E_1_X_1_X_2_E_2_X_3_, or X_1_X_2_X_3_E_2_X_4_, while GbNPF4.7 exhibits a complete motif replacement. Similarly, the E_1_X_1_X_2_E_2_[R/K] motif in the GbNPF7 subfamily is entirely substituted by QGLAT, which is also observed in the NPF7 subfamily of other plants [29].

The variability in the E1X1X2E2R motif, central to proton coupling and active transport, presents intriguing possibilities. In *Arabidopsis*, this motif has two variants, with arginine or lysine at the ends (E_1_X_1_X_2_E_2_R/E_1_X_1_X_2_E_2_K). The E_1_X_1_X_2_E_2_K motif is essential for glucosinolate transport in NPF2.11 [44]. Furthermore, AtNPF7.3, characterized by an entirely altered E1X1X2E2[R/K] motif, orchestrates potassium ion homeostasis by promoting the expression of potassium ion transporter under low nitrate conditions [45]. However, GbNPF5.8, GbNPF6.4 and GbNPF8.2 lack this motif. More research is needed to figure out their actual roles and how they transport nitrogen compounds. Our study unveils variations in this motif among GbNPF members, suggesting potential variations in their ability to transport protons and their functional diversity. This emphasizes the complex ways the NPF family works across different plants.

### Expression profile and cis-elements analysis revealed the potential role of *GbNRTs* in callus browning regulation

Browning is a major challenge in plant tissue culture. Ginkgo, a gymnosperm known for its flavonoid production, faces challenges in establishing regeneration and production systems due to browning [30, 46]. In this study, we treated zygotic embryos with varying nitrate concentrations during callus induction, revealing that higher nitrate concentrations resulted in deeper browning of Ginkgo calluses (Fig. 5). Many studies have indicated a connection between browning and nitrogen levels [47–49]. In *Camptotheca acuminata*, Zhang et al. [50] discovered that using Schenk & Hildebrandt (SH) basal salt is less likely to brown compared to using Murashige & Skoog (MS) basal salt. The SH basal salt contains a lower nitrogen concentration than MS. Daigen [33] observed that using reduced nitrogen compounds like KNO_3_ and NH_4_NO_3_ can decrease browning in rice. Moreover, Chopin found that the seed coat of the *nrt2.7* mutant in *Arabidopsis* exhibited brown coloring [28]. Ogawa et al. [51] hypothesized that low nitrite reductase activity could lead to high levels of nitrate, causing browning and stopping growth.

NPFs are responsible for transporting various substrates across cell membranes, such as nitrate, peptides, amino acid, and other organic compounds [52, 53]. In plants, NPFs play a critical role in nitrogen uptake and utilization, which is essential for plant growth, development, and stress responses [54]. Among the 53 NPF proteins in *A*rabidopsis, 20 have been shown to transport nitrate, while peptide transport activity has only been demonstrated in the NPF8 subfamily. In addition, NPF proteins could transport other substrates, such as nitrite [55], chloride [56], glucosinolate, auxin (IAA [57], 2,4-D [58]), abscisic acid (ABA), jasmonates (JAs) [59] and gibberellins (GAs) [60, 61]. This diverse range of functions highlights the importance of the NPF family in plant physiology, growth and development, and resistance to biotic and abiotic stresses.

Analysis of the promoter regions of the *GbNRTs* also revealed several hormone-related *CREs*. Among the 72 *GbNRTs* genes, 30 of their promoter regions contained Auxin-associated *CREs* (AuxRR-core, TGA-box, TGA-element) (Table S7). Expression analysis revealed that out of the differentially expressed 21 genes, 14 were higher expressed in calluses under LN treatment, and only 7 were higher expressed in calluses under HN treatment (Table S8). These findings suggest that auxin may affect the calluses' browning in Ginkgo. Previous studies have shown that adding 1-Naphthylacetic acid (NAA) promoted the proliferation of jasmine callus, increased the total phenol, flavonoid content, and free radical clearance, and inhibited browning compared to 2,4-D [62]. Additionally, studies in maize and Arabidopsis have exhibited that the inhibition of root growth under high nitrogen conditions is due to the decrease of root IAA level [63, 64], and root growth was promoted after switching from high nitrogen to low nitrogen conditions [64]. Furthermore, Krouk et al. [57] found that *Arabidopsis* NRT1.1 inhibited auxin accumulation at low nitrate concentrations. These results indicate that there is a signaling regulation between nitrogen and auxin, and nitrate may indeed affect plant growth and development by affecting auxin.

Enzymatic browning occurs when polyphenols, such as phenolic acids, flavonoids, and tannins, undergo oxidation [65, 66]. The synthesis of these polyphenols is regulated by transcription factors activated by various plant hormones, such as methyl jasmonate [67], abscisic acid [68], auxin [69], and ethylene [70]. The promoter region of GbNRTs contains numerous transcription factor binding sites, such as MYB and bHLH (Fig. 3), suggesting their potential role in regulating polyphenol synthesis. In red-fleshed apple, MdMYB10 is a key transcription factor that determines the fruit coloration [71, 72] and is associated with increased accumulation of anthocyanins due to enhanced nitrate uptake through the activation of MdNRT2.4–1 [73]. However, recent research has shown that brassinolide inhibits flavonoid biosynthesis and coloration in apples through a MdBEH2.2-MdMYB60 complex [74]. In Arabidopsis, the interaction between Teosinte branched1/ Cycloidea/ Proliferating cell factors (TCP) protein and V-myb avian myeloblastosis viral oncogene homolog (R2R3-MYB) protein promoted flavonoid biosynthesis while negatively regulating the auxin response [75]. Overall, the regulation of polyphenol synthesis is a complex and dynamic process that involves multiple factors and pathways.

Our findings suggest that GbNPFs, acting as nitrate sensors and transporters, influence the signaling pathways of nitrate uptake and assimilation, as well as auxin and polyphenol biosynthesis, to control browning during callus induction in Ginkgo. However, additional research is necessary to explore the mechanisms through which nitrates impact phenolic substance formation and how nitrates and auxins influence plant browning.

## Conclusions

In this study, we aimed to explore the potential role of GbNRTs in browning during callus induction in Ginkgo by analyzing their evolutionary conservation, functional diversity, and expression profiles. We identified 74 GbNRTs, including 68 GbNPF, 4 GbNRT2, and 2 GbNRT3, and found that high nitrate concentrations deepened the browning during callus induction in Ginkgo. Our analysis of the promoter region and expression profiles of calluses under LN and HN conditions suggest that GbNRTs play roles in orchestrating nitrate uptake and assimilation, as well as auxin and polyphenol biosynthesis, to control browning in Ginkgo. This study provides a foundation for further research on the effects of NO_3_^−^ and NRTs genes on browning in vitro culture.

## Materials and methods

### Plant materials and treatments

The fruits of *G. biloba* (Taixing No. 4) were harvested on October 3, 2021, from the Ginkgo Germplasm Resource Garden in Pizhou, Jiangsu Province. Zygotic embryos were dissected are cultured in the medium containing modified Gupta and Durzan basal salt (DCR) with varying NO_3_^−^ [76] and supplemented with 0.2 mg/L 2,4-D, 0.05 mg/l TDZ (Table S9). Each treatment utilized 30 zygotic embryos with three biological replicates. The embryos were then incubated under a light intensity of 6000 lx, photoperiod 16/8 h, and a temperature of 25 ± 2℃. Calluses induced from the zygotic embryos were harvested at 17 DAC and 26 DAC, flash-frozen in liquid nitrogen, and stored at -80 ℃.

Identification of the Ginkgo *NRT* genes and prediction of amino acid characteristics.

To identify putative NRT protein sequences in *G. biloba*, 53 NPF sequences, 7 NRT2 sequences, and 2 NAR2 sequences of *A. thaliana* were obtained from the TAIR website (https://www.arabidopsis.org/). The Ginkgo genome was downloaded from the Genome Sequence Archive (GSA) platform (CRA002041). The Arabidopsis NRT sequences were used as queries to search for homologous sequences in the Ginkgo genome using the blast function in TBtools [77]. The putative sequences were submitted to the Pfam database (http://pfam.xfam.org/) to determine the presence of the PTR2 (PF00854) core domain of the NPF subfamily, the MSF_1 (PF07690) core domain of the NRT2 subfamily, and the NAR2 (PF16974) core domain of the NRT3 subfamily. The integrity of the conservative domains was verified using the batch CD-search tool at the National Center for Biotechnology Information (NCBI) (https://www.ncbi.nlm.nih.gov/Structure/bwrpsb/bwrpsb.cgi). GbNRT proteins with less than 200 amino acid residues and 40% of PTR2 domains missing were removed from the analysis, and the remaining sequences were considered functional and used for further analysis.

To predict the transmembrane regions and subcellular localization of the putative GbNRTs, the TMHMM-2.0 (https://services.healthtech.dtu.dk/service.php?TMHMM-2.0) and the WoLF PSORT tool (https://wolfpsort.hgc.jp/) were used, respectively. The ProtParam tool of the ExPASy program (https://web.expasy.org/protparam/) was used to calculate various physicochemical properties of the putative GbNRTs, including molecular weight, theoretical isoelectric point (pI), instability index, grand average of hydropathicity (GRAVY), etc.

### Phylogenetic analysis and structural characterization of GbNRTs

The full-length sequences of NRTs proteins from *A. thaliana*, *P. trichocarpa*, *P. pinaster* and *G. biloba* were aligned using the MUSCLE tool. The neighbor-joining (NJ) method was used to construct rootless phylogenetic trees with the MEGA7.0 software [78]. The bootstrapping method with 1000 repetitions was employed to assess the robustness of the trees. The NRT sequences of *A. thaliana, P. trichocarpa* and *P*. *pinaster* were obtained from previous reports [10] (Table S1), and the GbNRTs were named according to the nomenclature recommended by Léran et al. (2014). All GbNRTs protein sequences were checked and analyzed for conserved domains using NCBI CD-Search tool. The MEME Version 5.5.0 (https://meme-suite.org/meme/tools/meme) was used for conservative motif analysis and the maximum cardinal number was set to 10 for NPF and NRT2, and 4 for NRT3. The gene structure annotation file from the Ginkgo genome release was used for gene structure visualization.

### Chromosomal localization and collinearity analysis of GbNRTs

The locations of the 74 *GbNRTs* genes were obtained from the Ginkgo Genome Database and visualized using TBtools software to show their position on chromosomes. Gene repeat events and collinearity relationships were analyzed using the Multiple Collinear Scan Toolkit (MCScanX) in TBtools. Collinearity circs plot of the NRTs gene in the Ginkgo genome and collinearity map between different species were constructed. The Ka/Ks calculator program in TBtools calculated the non-synonymous substitution rate (Ka), synonymous substitution rate (Ks), and Ka/Ks between tandem repeats pairs and paralogs.

### Analysis of *CREs* and transcription factor binding sites in the promoter region of GbNRTs

The 2000 bp upstream of the transcription start site (ATG) of the *GbNRT* genes was extracted from the ginkgo genome file as a promoter region. PlantCare (https://bioinformatics.psb.ugent.be/webtools/plantcare/html/) was used to detect *CREs*, and PlantTFDB v5.0 (http://planttfdb.gao-lab.org/prediction.php) was used for predicting transcription factor binding sites. Data analysis and graphing were performed using Excel.

### Transcript abundance analysis

Transcriptome sequencing was performed to detect the genome-wide expression of *GbNRTs*. Based on the RNA-seq data, the transcriptional abundance of *GbNRTs* was calculated using FPKM (Fragments per Kilobase of Exon per million reads Mapped) values. Microsoft Excel 2016 was used to convert (FPKM + 1) to log base 2, and TBtools (TBtools.v1.09854) was used to create a heat map.

### Expression of *GbNRTs* via qRT-PCR

The total RNA was extracted using Easy Plant RNA Kit (DR0407050, Easy-Do make it easier, Zhejiang, China) and stored at − 80 °C until further use. The purity and integrity of the isolated total RNA were evaluated using agarose gel electrophoresis and the NanoDrop™ One/OneC trace UV–Vis spectrophotometer. cDNA was synthesized using cDNA Synthesis Kit PrimeScript™ RT Master Mix (Perfect Real Time) (RR036A, Takara, Beijing, China). qRT-PCR was performed on CFX96 Touch Real-Time PCR Detection System using ChamQ Universal SYBR qPCR Master Mix (Q711-02, Vazyme, Nanjing, China). The *GbUBQ* gene was the reference gene. The primer sequences used are listed in Table S10. The relative expression levels of genes were calculated using the 2^−ΔΔCt^ method.

### Supplementary Information

**Additional file 1.**

**Additional file 2.**

**Additional file 3.**

**Additional file 4.**

**Additional file 5.**

**Additional file 6.**

**Additional file 7.**

**Additional file 8.**

**Additional file 9.**

**Additional file 10.**

**Additional file 11.**

**Additional file 12.**

**Additional file 13.**

## Data Availability

All data used in this study are included in this article and additional files. The Genome sequence and annotation datasets are available in: (https://ngdc.cncb.ac.cn/gsa/browse/CRA002032) and (https://ngdc.cncb.ac.cn/gsa/browse/CRA002041).All the genes used in this study for phylogeny and subsequent analysis are mentioned in additional file 1, table S1. Transcriptome data used for gene expression analysis are mentioned in Additional file 9, table S6.

## Abbreviations

LATS: Low-affinity transport system
HATS: High-affinity transport system
DATS: Dual-affinity transport system
TMs: Transmembrane domains
aa: Amino acids
*CREs*: *Cis*-Acting regulatory elements
TF: Transcription factor
DAC: Days after cultivate
LN: Low nitrate condition
HN: High nitrate condition
IB: Inhibiting browning
PB: Promoting browning
2,4-D: 2,4-Dichlorophenoxyacetic acid
TDZ: Thidiazuron
