# Supplementary material for: Genome-wide identification and expression analysis of the NRT genes in Ginkgo biloba under nitrate treatment reveal the potential roles during calluses browning
Source: BMC Genomics. 2023 Oct 23;24:633. doi: 10.1186/s12864-023-09732-4 (PMC10594704; doi:10.1186/s12864-023-09732-4)
Supplement: Supplementary file 13 — Additional file 13. [file 12864_2023_9732_MOESM13_ESM.pdf]

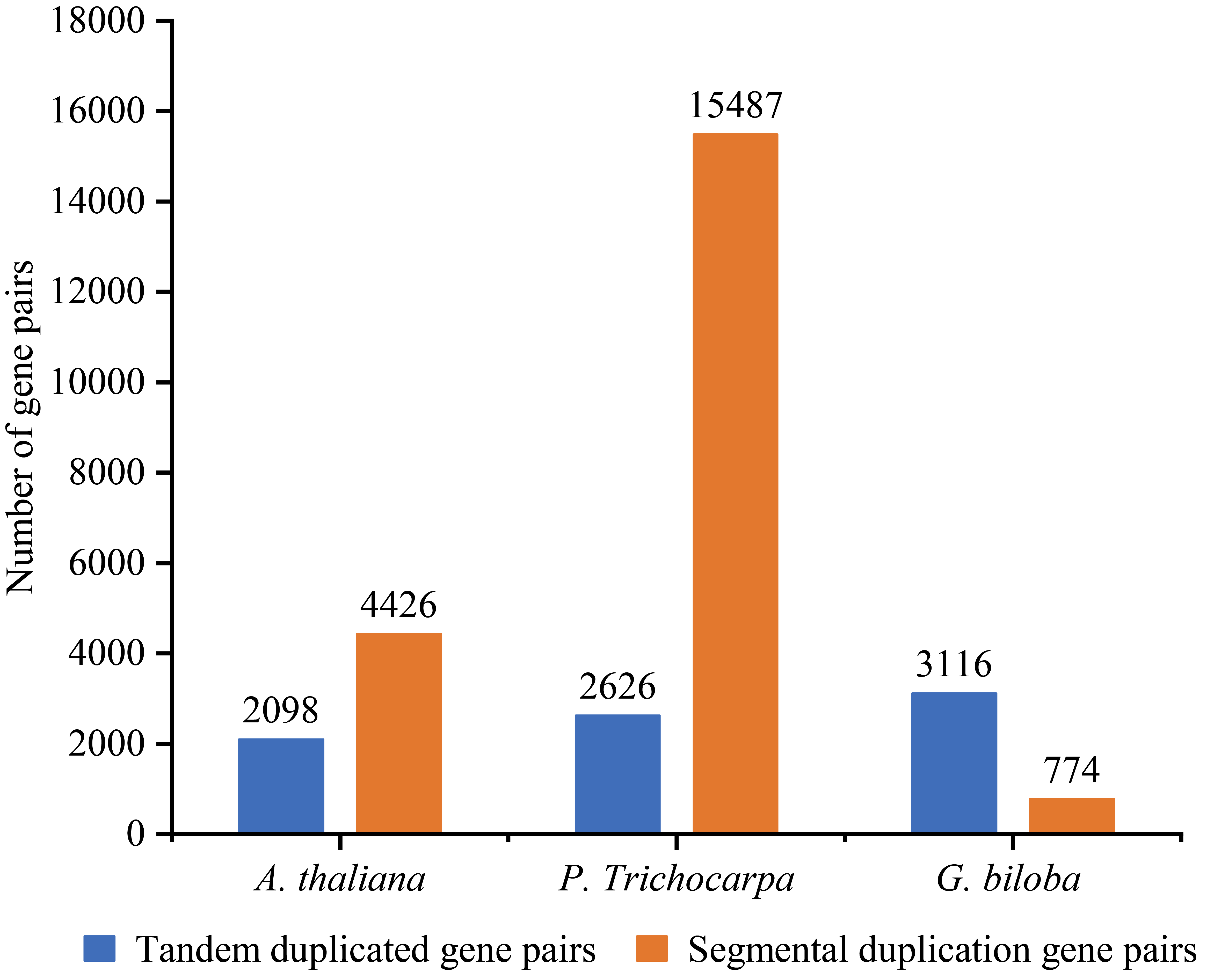

Figure S3. The number of gene pairs produced by different replication events of *A. thaliana*, *P. trichocarpa* and *G. biloba*.
