# Supplementary material for: Genome-wide identification and expression analysis of the NRT genes in Ginkgo biloba under nitrate treatment reveal the potential roles during calluses browning
Source: BMC Genomics. 2023 Oct 23;24:633. doi: 10.1186/s12864-023-09732-4 (PMC10594704; doi:10.1186/s12864-023-09732-4)
Supplement: Supplementary file 12 — Additional file 12. [file 12864_2023_9732_MOESM12_ESM.pdf]

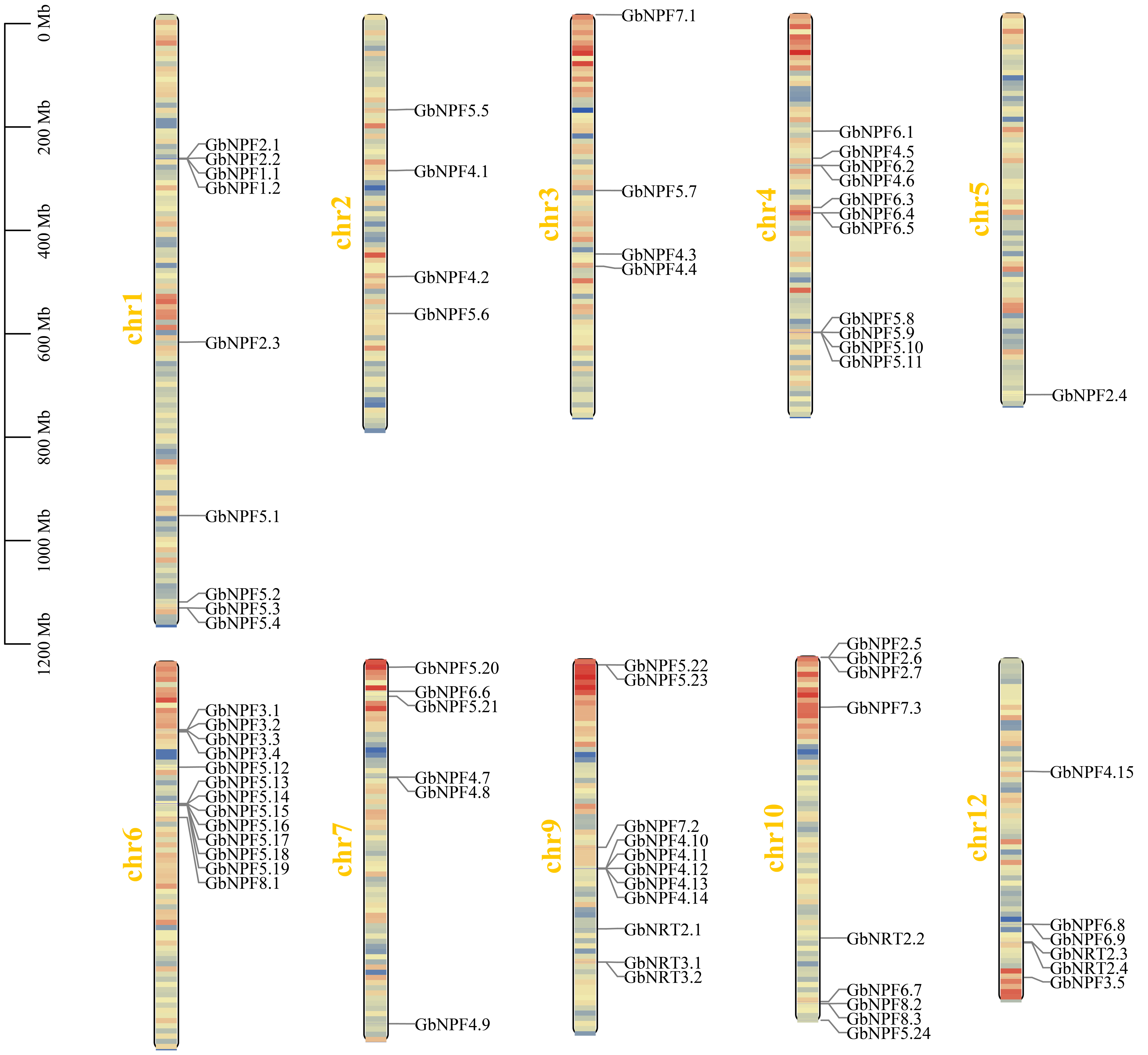

Figure S2. Chromosomal locations of *GbNRTs* in *G. biloba* genome.

The length of the chromosomes is labeled, and the density of genes on the chromosomes is calculated. With 1Mb as 1 unit length, the blue indicates that the number of genes per unit length is less, and the red indicates that there are more genes per unit length.
